# Evolutionary reversion of live viral vaccines: Can genetic engineering subdue it?

**DOI:** 10.1093/ve/vev005

**Published:** 2015-07-31

**Authors:** J. J. Bull

**Affiliations:** ^1^Department of Integrative Biology, The University of Texas at Austin, Austin, TX 78712, USA; ^2^Institute for Cellular and Molecular Biology, The University of Texas at Austin, Austin, TX 78712, USA; ^3^Center for Computational Biology and Bioinformatics, The University of Texas at Austin, Austin, TX 78712, USA

**Keywords:** virus, synthetic biology, *R*_0_

## Abstract

Attenuated, live viral vaccines have been extraordinarily successful in protecting against many diseases. The main drawbacks in their development and use have been reliance on an unpredictable method of attenuation and the potential for evolutionary reversion to high virulence. Methods of genetic engineering now provide many safer alternatives to live vaccines, so if live vaccines are to compete with these alternatives in the future, they must either have superior immunogenicity or they must be able to overcome these former disadvantages. Several live vaccine designs that were historically inaccessible are now feasible because of advances in genome synthesis. Some of those methods are addressed here, with an emphasis on whether they enable predictable levels of attenuation and whether they are stable against evolutionary reversion. These new designs overcome many of the former drawbacks and position live vaccines to be competitive with alternatives. Not only do new methods appear to retard evolutionary reversion enough to prevent vaccine-derived epidemics, but it may even be possible to permanently attenuate live vaccines that are transmissible but cannot evolve to higher virulence under prolonged adaptation.

## 1 Introduction

DNA technology has advanced, so that it is now feasible to reconstitute the genomes of many organisms on at least a small scale and, in some cases, a large scale. This technology allows us to create new gene sequences almost at will, to possibly resurrect extinct species, and even to create functions and organisms that never existed. Applications seem almost limitless, spanning engineered vaccines, foodstuffs with improved nutrition, biofuels, industrial enzymes, nanoscale electronics, living sensors, and transforming insects to incapacitate their ability to transmit diseases.

In some contexts, the challenge of genetic engineering goes beyond synthesis and function. Any self-replicating genome is subject to evolution, and if that genome is grown as a population of individuals—as it must be in many of these applications—evolution may reverse the engineering toward unwanted ends. The second challenge of genetic engineering is thus to predict that evolution and take measures to avoid or reduce it. Yet predicting evolution represents its own set of difficulties, and designing genomes to avoid that evolution poses others.

This article considers the evolution of engineered genomes for use as live, attenuated viral vaccines. These vaccines consist of infectious viruses that establish a limited infection in the patient, mimicking infection by the wild-type virus enough to elicit an immune response but not enough to cause disease. They may also be transmitted to people other than those vaccinated. The attenuated viral populations created in this fashion, whether within or among patients, are thus subject to natural selection to improve viral growth rate. Any consequent evolution is usually antithetical to their intended use, possibly reversing the attenuation. The purpose of this article is to consider some of the major ways to engineer live vaccines, especially with respect to the ability to limit this post-engineering evolution. Viral evolution is not an issue with killed vaccines or with subunit vaccines in which the vector cannot create infectious virus in the host, so those types of vaccines are not considered here.

## 2 The perspective

Live vaccines have been useful, but existing ones are not without risk. Poliovirus is a good example. The oral polio vaccine (OPV) consists of three viral strains, each attenuated for low virulence. The risk of disease from the vaccine is maybe 1 in 750,000 recipients, and the immunity is lifelong (Burns et al. 2014). This vaccine was responsible for polio eradication in the Americas and Europe (Nathanson and Kew 2010; Kew 2012). But one serotype is only two mutations away from attenuation reversal, another is three mutations away. In countries with intermittent vaccine coverage, the vaccine has evolved reversal of the attenuation many times, causing epidemics and even establishing itself endemically (Kew 2012). With current political constraints, a safer OPV that does not easily revert yet still stimulates mucosal immunity would be highly desirable. Can synthetic biology give us a better live vaccine?

The pursuit of a better live vaccine must be considered in light of new alternatives. Many new methods for creating vaccines exist, some which combine benefits of older methods without the drawbacks. In particular, there are now various ways of creating virus particles that are not infectious or virions that can infect a cell and go through one intracellular life cycle yet produce no infectious progeny (Chroboczek, Szurgot, and Szolajska 2014; Yamanaka, Suzuki, and Konishi 2014; Petro et al. 2015). DNA vaccines can express subsets of the viral proteome without risk of producing infectious particles (Liu 2011). These and other ‘dead-end’ vaccines provide novel ways of exposing the patient to large repertoires of viral proteins in relevant tissues with zero risk of creating a replicating, sustained viral population in the patient. There is consequently no possibility of vaccine escape to secondary contacts, no possibility of a vaccine-derived epidemic.

Live vaccines do have advantages over these non-infectious alternatives, however (Lauring, Jones, and Andino 2010). By establishing a self-replicating infection, the vaccine inoculum can be much lower than with the dead-end approaches. The resulting immunity may be greater. Also, if the vaccine strain is transmitted to secondary contacts without reverting to higher virulence, herd immunity will increase passively (Hanley 2011), reducing the public health effort needed to achieve the same level of coverage with dead-end vaccines. Transmissible vaccines even have the potential to reach populations that other approaches cannot, such as wildlife and inaccessible humans. Attenuated viruses might also be used in the production of killed-virus vaccines, as the killing is not invariably complete, and the attenuation would help prevent disease (as suggested by a reviewer).

Synthetic biology not only offers new alternatives for the development of dead-end vaccines, it also enables new approaches to the design of live virus vaccines. For these new approaches to be justified against the non-replicating alternatives, two problems must be solved. First, the level attenuation should be tunable, so that the virus is neither virulent nor so incapacitated that it fails to generate a sufficient immune response. Second, reversion to virulence must be so slow that the descendants of the vaccine disappear before evolution enables it to initiate an epidemic. Both of these objectives will be greatly facilitated by attenuation methods that lend themselves to predictability. Such is the focus here.

## 3 Population dynamics and escape of a vaccine strain

The reversal of an attenuated vaccine strain is an evolutionary process. It is an unusual evolutionary process because it occurs in a population declining toward extinction—a vaccine strain that has been introduced into patients, where developing immunity will quickly extinguish the infection. The goal is to ensure that the attenuated virus dies out after each administration. It is not necessary to block all evolution of the vaccine strain, only that any evolution be slow enough and small enough to still allow extinction.

The evolutionary issues of a vaccine strain are conveniently explained in terms of the basic reproductive of the infection, *R*_0_, which is the ‘fecundity’ of a single infected host in a naive population—the number of new hosts infected (Antia et al. 2003). Attenuated strains are designed either to be confined to the vaccinated individual, thus failing to transmit entirely ($R_{0}=0$), or to transmit so poorly that each vaccinated host establishes fewer than one new infection on average ($0<R_{0}<1$). However, the growth of attenuated viral populations within patients and among patients allows the ascent of mutations that increase *R*_0_, and if *R*_0_ evolves to exceed 1, the vaccine strain can potentially create an epidemic. Killed virus, designs that create empty, virus-like particles, and dead-end viruses that infect a cell but produce non-infectious progeny necessarily have $R_{0}=0$, but even live vaccines that produce infectious progeny may also fail to transmit to new patients because they do not establish a high enough titer.

$R_{0}=1$ is known as the epidemic threshold, because a virus that exceeds the threshold can start an epidemic in a purely naive population. That is, $R_{0}>1$ defines conditions necessary for viral persistence. Even if a vaccine strain evolves $R_{0}>1$, it need not persist—herd immunity in the population may prevent its spread. Yet although $R_{0}>1$ is not a sufficient condition for allowing viral persistence, preventing evolution of $R_{0}>1$ is at least a conservatively safe goal for a live vaccine and may even be necessary at the brink of eradication.

**Figure 1.**
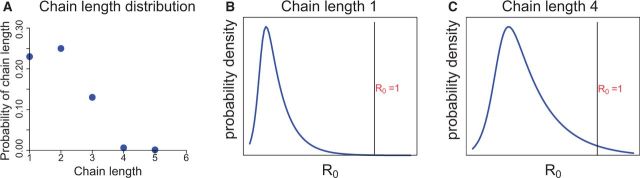
Hypothetical, visual model for evolution of an attenuated virus to exceed the epidemic threshold. (A) For each individual vaccinated, there is a distribution of average transmission chain lengths that depends on the number of secondary contacts infected and how many contacts they infect. Thus, the vaccinated individual may transmit to (a) no one (chain length 1), (b) to one secondary contact but they fail to transmit (length 2), (c) to two secondary contacts, one of which does not transmit further (length 2) and one of which transmits to a single contact who does not transmit (length 3), and so on for all possibilities. The average chain length increases with the average *R*_0_ of the virus. (B and C) For the viruses that have attained a particular chain length, there will have been some evolution occurring during that chain, resulting in a distribution of *R*_0_ values increasing with chain length. If the chain is long enough, there may have been enough evolution that some viruses now have $R_{0}>1$, and if they get into the next patient, an outbreak may occur (depending on other factors). The net probability of evolving a virus that exceeds the epidemic threshold combines the probability of each chain length with the probability that viruses in that patient have $R_{0}>1$ and escape to the next patient. The net probability of escape will be vanishingly small if chain lengths are short and the virus either never evolves $R_{0}>1$ or is slow to do so.

The probability that a vaccine strain evolves above the epidemic threshold depends on transmission chain length and the probability that viruses at that stage of transmission will have the mutations necessary for $R_{0}>1$ (Fig. 1). As with any adaptive evolutionary process, the magnitude of evolution is expected to increase with population size and with increasing time to extinction. Vaccines whose intrinsic *R*_0_ (before evolution) are 0.4 or less are expected to die out quickly (they have a very low probability of transmission chains of 4 or more from a single infected individual), whereas those of $R_{0}>0.6$ have a much higher probability of long chains (Farrington and Grant 1999). Viruses with an *R*_0_ near 1 are especially prone to evolve above the epidemic threshold both because they are likely to experience long transmission chains and because they need little increase in *R*_0_ to exceed the threshold (Antia et al. 2003). Based on population size considerations, it might also seem that adaptive vaccine evolution increases directly with the number of individuals vaccinated, but herd immunity generated by mass vaccination will reduce opportunities for transmission and thus restrict evolution.

*R*_0_ is an average measure, and as such, it may conceal potential problems. With some viruses, including poliovirus, an occasional vaccinated individual establishes a persistent infection, perhaps due to a defect in their immune system (Adams et al. 2012; Burns et al. 2014). These individuals may transmit the virus to far more secondary contacts that does the average vaccinated person, and they may even be incubators for extensive, long-term vaccine evolution within the host (Baric et al. 1999; DeVries et al. 2011). If these hosts are uncommon, their presence will have almost no impact on standard *R*_0_ calculations, yet they alone could be the basis of vaccine evolution to $R_{0}>1$. Nonetheless, it would be useful to know the *R*_0_ of live, attenuated viruses. Such studies may require new estimation approaches, because mass vaccinations exhibit different dynamics than do natural outbreaks. Yet for some viruses, the estimations may be facilitated by easily observed, population-wide decay in viral shedding after vaccinations (e.g., Troy et al. 2014).

In addition to the impact of evolution on *R*_0_, an equally important consideration is the impact on virulence. An increase in *R*_0_ does not necessarily translate into increased virulence, but there is a general sense that increases in *R*_0_ ultimately lead to increases in virulence, if only because the reverse applies—attenuation results from sufficiently reduced viral growth (Lauring, Jones, and Andino 2010). Understanding how virulence and *R*_0_ are coupled for any virus would be most welcome, because in the absence of such knowledge, there is *a priori* no way to know what reduction in viral growth rate is sufficient for disease absence (Meng et al. 2014).

**Figure 2.**
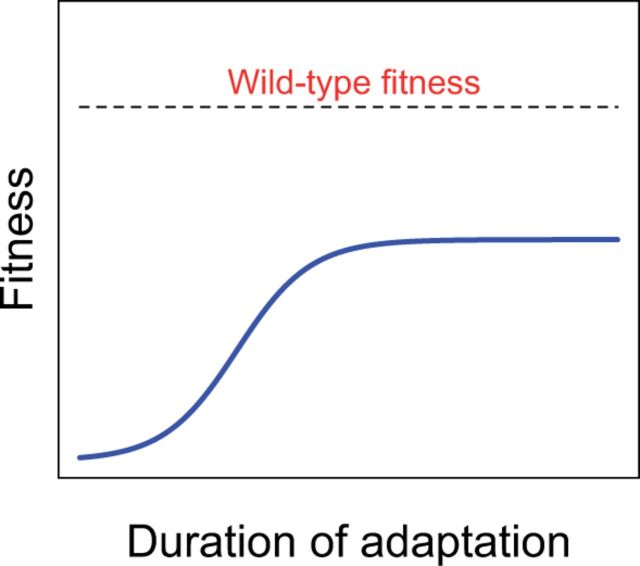
A visual model of permanent attenuation. Fitness during continued evolution of the engineered virus (blue curve) reaches a plateau below that of the wild-type virus (dashed).

Some attenuation designs may so constrain evolution that the virus is unable to evolve $R_{0}>1$ (Fig. 2) or is unable to evolve higher virulence despite $R_{0}>1$. Such designs would allow the vaccine to be used without fear of reversion. A design of permanent attenuation with $R_{0}>1$ raises the possibility of extinguishing a wild-type virus and then maintaining a self-perpetuating, transmissible vaccine that would suppress any reintroduction of the wild type. Synthetic biology should enable us to create ‘evolution-proof’ vaccines, but doing so requires that we understand how reversion evolves.

## 4 General attenuation methods

The polio example is one of many that motivates new methods of vaccine design. The challenge of poliovirus is unusual in that it is a case in which we can no longer afford reversion to wild-type virulence. Yet vaccine reversion becomes an issue even for viruses eradicated on a local scale. This section describes several attenuation methods, with a strong emphasis on designs enabled by genome synthesis. The coverage here is not comprehensive (e.g., viral chimeras are omitted, as are many methods specific to genetic details of a particular virus) but instead emphasizes methods with potential generality across many different viruses, where there is some anticipation of prediction, and for which some data on reversion are available. The framework presented here is easily extended to methods not covered.

To anticipate points made below, it should be apparent that the evolutionary reversal of an attenuated strain, leading to an increase in *R*_0_ and possibly in virulence, does not require an actual genetic reversal of the attenuating mutations. The reversing mutations may leave intact the attenuating mutations and merely compensate for their effects, enabling a fitness increase. Thus the design of an attenuated strain to avoid strict genetic reversal of the changes does not ensure the absence of an evolutionary reversal of the attenuation.

## 5 The old way: haphazard, no control over attenuation or recovery

The standard method for viral attenuation, the one used for at least half a century, is haphazard (Fenner and Cairns 1959; Lauring, Jones, and Andino 2010; Hanley 2011). The foundation of this method, and indeed nearly all methods of attenuation, is to create a genetic derivative of the disease-causing, wild-type virus that grows poorly in the host. The old method uses viral growth to evolve a virus that is genetically ‘repressed’ from its wild counterpart. Toward this end, the virulent, wild-type virus is propagated under unnatural conditions such that it has an opportunity to adapt to the new environment. The new environment may be a tissue culture with a sub-optimal temperature or cells of an unnatural host, or the new environment may be the unnatural host itself. Any adaptation to the new conditions may render it less able to grow well in the original host—an example of what is commonly referred to as an evolutionary trade-off or a negative genetic correlation.

This method is haphazard because every set of growth conditions is potentially different, and even different attenuations of the same virus using the same protocol may follow different trajectories. The resulting strain may be a mix of genetic variants (Badgett et al. 2002; Depledge et al. 2014); plaque purification may resolve it to a single genotype, but not all viruses can be grown as plaques. Prior to testing, there is no basis for knowing whether adaptation to the new environment affects viral ability to grow in the original host—predicting the level of attenuation is impossible. Thus, any candidate vaccine must be tested by standard trial and error with a susceptible host. In the case of poliovirus, that host was the chimpanzee: a vaccine candidate was injected into its cerebral spinal fluid and the chimp monitored for disease. Although chimps were not natural hosts for poliovirus, wild-type virus delivered in this fashion could make them sick. For many other cases, the virus must be tested in humans before attenuation is known.

Predicting the potential for vaccine reversion is also problematic with this method. When the method was applied before DNA and RNA sequencing became routine, there was no way to know how many mutations were acquired in the attenuation process. Easy reversion of poliovirus might have been anticipated from the realization that attenuation was accompanied by few mutations. However, the use of sequencing may not provide a definitive answer: a low mutation count in the attenuated strain may forebode easy reversion, but a high mutation count does not ensure slow reversion because most of the mutations may be irrelevant to attenuation. Even more problematic, attenuation by this method may yield genetically polymorphic strains, and reversion to high virulence may involve variants at low frequencies, something not easily identified or interpreted from sequences (Badgett et al. 2002; Todd et al. 2003; Depledge et al. 2014).

Closely related to attenuation by unnatural growth is the use of a viral relative for attenuation, the classic example being Jenner’s use of cowpox to immunize against smallpox (Snchez-Sampedro et al. 2015). Attenuation by this method is also haphazard, especially considering that even different serotypes of the same virus may not provide cross protection. However, a virus from another host may be many mutations away from higher virulence.

## 6 Engineered attenuation by silent codon changes: control of fitness and recovery

It has long been known that the fixation of random mutations in a population will lead to a decrease in average population fitness, and a simple way to fix random mutations is to force the population through a series of extreme bottlenecks (Chao 1990; Elena et al. 2001; Bull et al. 2003). Methods of genetic engineering would allow us to synthesize genomes with random mutations more expeditiously than by sequential bottlenecks, enabling us to create and test hundreds of mutant genomes for any virus. As an attenuation method, however, the fixation of random mutations poses problems. First, the fitness effects of random mutations vary greatly, so the fitness decline from, say, 10 random mutations might be due primarily to just one or two of those mutations, potentially allowing for easy recovery (Sanjuan, Moya, and Elena 2004; Domingo-Calap, Cuevas, and Sanjuan 2009). Second, mutations that alter amino acid sequences may change the antigenicity of the vaccine, so that immunity is no longer targeted directly at virulent forms of the virus.

Two articles in 2006 proposed attenuating poliovirus by introducing point mutations in a way that overcomes both problems (Burns et al. 2006; Mueller et al. 2006). The suggestion was to create a viral genome with altered codon sequences but which encodes protein sequences identical to the wild type. To a first approximation, a silent codon substitution makes little difference. The protein is the same (hence the codon changes are ‘silent’). But some exchanges do make small fitness differences, so they are not strictly fitness neutral. If many exchanges are made in a viral genome and the exchanges are of the right type, viral reproduction can be slowed down to arbitrary levels yet the virus encodes the same proteins as the wild type and is antigenically the same as wild type. Advantages of this method are that it can potentially be used with any virus, even those with small genomes (Tulloch et al. 2014), and no detailed knowledge of viral biochemistry is required. Other methods discussed below are not as obviously feasible with small genomes.

There are several mechanisms by which a silent codon change can affect fitness, and understanding those mechanisms is important both for achieving attenuation and in limiting evolutionary reversion. By choosing rare codons, protein translation may be slowed (slowing viral progeny production) and translational error rates may be increased (leading to misfolded proteins). RNA secondary structure may be altered by silent codon changes, and regulatory signals may be created or destroyed. Also, dinucleotide frequencies will often be altered. Three rules for making the ‘right type’ of silent codon exchanges have been proposed, all implemented in experimental poliovirus studies. One method substitutes rare codons for common ones (Burns et al. 2006; Mueller et al. 2006). Another merely shuffles the synonymous codons used by the gene (Coleman et al. 2008). A third specifically introduces CpG and UpA dinucleotides (Burns et al. 2009). There are varying degrees of overlap in the different methods (e.g., codon shuffling does not introduce rare codons but it does affect CpG and UpA dinucleotides), but the bulk of evidence seems to support a mechanism of attenuation by introducing CpG and UpA dinucleotides (Atkinson et al. 2014; Tulloch et al. 2014, see below).

### 6.1 Prediction

Large-scale, silent codon modification of a viral genome appears to be an easy and predictable way to attenuation: if each engineered codon change has a small individual effect, achieving a significant magnitude of attenuation will require many such changes and the level of attenuation will be finely adjustable. The method also offers a second predicted advantage for a vaccine: slow reversion. In theory, if hundreds of codon changes individually contribute small effects to reduced viral growth, the pathway to viral recovery must reverse most of those changes. Any single reversion mutation should ascend slowly because of its small fitness benefit. The most likely large-scale reversion process imaginable under this model is the protracted accumulation of many individual mutations (Burns et al. 2006; Mueller et al. 2006; Coleman et al. 2008; Burns et al. 2009). Reversion not only seems as if it must be incredibly slow but is even prone to evolve in directions that do not recover wild type, possibly getting trapped on fitness peaks below that of wild type.

The prediction of slow recovery could fail for two reasons. First, if the fitness effect size of different silent codon changes is highly variable, then possibly few codon reversals would enact a substantial recovery. The fact that fitness declines approximately linearly with number of altered codons in some implementations (see below) would seem to argue against this, at least for those viruses. Second, the argument of slow fitness recovery assumes that evolution must reverse each of the attenuating mutations to reverse the attenuating fitness effect. This assumption is more suspect. Recovery could be due to mutations that lie outside the attenuating changes, such as those that change the balance of gene expression across the genome. The fitness effects for those types of changes need not obey the predicted incrementality of the evolutionary process.

### 6.2 Empirical tests

**Figure 3.**
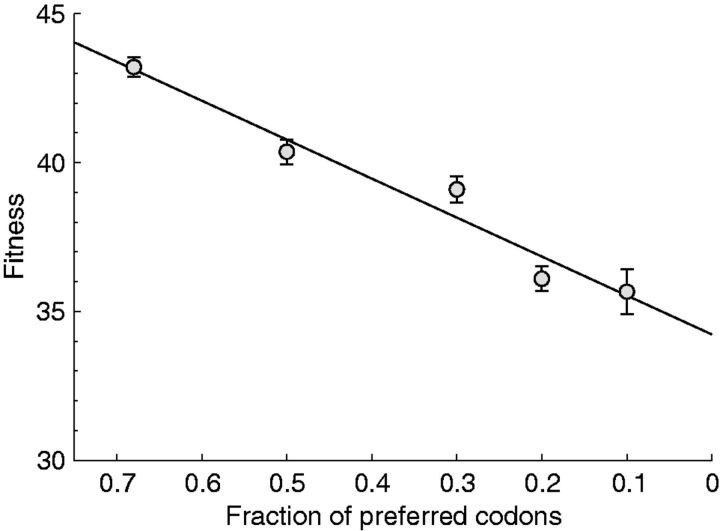
Fitness declines approximately linearly with the number of suboptimal codons in phage T7. All changes were engineered in the viral major capsid gene. Fitness is growth rate, population doublings per hour, a geometric measure of fitness. The leftmost point represents wild type. Used with permission from Bull, Molineux, and Wilke (2012).

Tests of the attenuation method and evolutionary recovery are sometimes limited to virus grown in culture (Burns et al. 2006, 2009; Bull, Molineux, and Wilke 2012; Martrus et al. 2013; Tulloch et al. 2014), though not always (Coleman et al. 2008; Mueller et al. 2010; Meng et al. 2014; Ni et al. 2014; Nogales et al. 2014; Noun et al. 2014; Wang et al. 2015). All three protocols proposed for poliovirus achieved attenuation in a satisfying, quantitative fashion (Burns et al. 2006, 2009; Mueller et al. 2006; Coleman et al. 2008). In a couple other systems as well, silent codon replacements have revealed highly regular, quantitative attenuation in proportion to the number of codon changes (Fig. 3, from Bull, Molineux, and Wilke 2012; Nougairede et al. 2013).

A recent proposal, extending the suggestion of Burns et al. (2009), based on a reanalysis of other studies and also on new data is that the level of attenuation for eukaryotic viruses depends chiefly on the number of CpG and UpA dinucleotides created by the codon changes. Rather than impairing translation, the attenuating effect is due to the innate immune response to the nucleic acid itself (Atkinson et al. 2014; Tulloch et al. 2014). If the dependence of attenuation on those two dinucleotides holds broadly, it will enable greater predictability in attenuation. This model cannot explain attenuation with bacterial viruses, of course. Given the many processes that could be affected by silent codon differences (protein synthesis, RNA folding, or translational errors), it is possible that, across all viruses, there are multiple mechanisms contributing to attenuation by silent codon replacement.

How well does do codon-modified viruses resist reversion when given a chance to evolve? In some short-term adaptations, there has been no obvious evolutionary fitness reversion (Coleman et al. 2008; Meng et al. 2014). In cases of longer term evolution, fitness reversion is slow or substantial—not consistent across viruses. Part of the difficulty in comparing results across studies is that some studies codon-modify viral genomes that have not been pre-adapted to the growth conditions used in the adaptations. Thus, both the codon-modified virus and the unmodified virus show considerable fitness gains during the adaptations, so it is difficult to know how much of the fitness improvement in the codon-modified virus is in response to the attenuation rather than the culture conditions. When fitness increase can be attributed to attenuation reversal, however, the nucleotide changes commonly do not reverse the attenuating mutations and may even fall outside the codon-modified segments (Bull, Molineux, and Wilke 2012; Martrus et al. 2013; Nougairede et al. 2013). Poliovirus reversions did show an elevated rate of CpG reversals (which led to the proposal of their importance in attenuation, Burns et al. 2009). Thus, molecular reversal of attenuation is not one to one with the engineered changes, and the fitness effects of reversing mutations are not as small as predicted. Some of the more rapid reversions were in HIV-1, with complete recovery of fitness in 15 passages (10-fold dilutions each, Martrus et al. 2013). Moderate fitness increases occurred in deoptimized Chikungunya virus over 300 generations, but inference from analysis of mutations was that most of the fitness gain was from adaptation to culture conditions, hence slow reversion of the engineered fitness drop (Nougairede et al. 2013). No detectable fitness increase was observed after 100 generations in phage T7, but nearly half of lost fitness was recovered after 1,000 generations (Bull, Molineux, and Wilke 2012).

Overall, evolutionary reversals of codon-deoptimized viruses sometimes exhibit a slow or moderately slow recovery but faster than predicted if reversion occurred by serially reversing the many mutations of individually small fitness effects. If CpG and UpA dinucleotides are the primary determinants of attenuation, the ability to predict recovery could improve, but a better understanding of the molecular mechanisms of attenuation may first be required. At present, experimental work is needed to determine the pace of recovery for each individual implementation. It nonetheless appears that the pace of recovery is potentially slow enough to avert vaccine escape for many viruses, provided the initial *R*_0_ is not close to 1. It is questionable, however, whether this form of attenuation is potentially permanent.

## 7 Engineered attenuation by genome rearrangement: toward permanent attenuation of an infectious virus?

The order of elements in a viral genome has major effects on its growth rate, through regulation, timing of gene expression, and possibly packaging signals. Furthermore, gene order is highly conserved in many viruses, suggesting that gene order is important to fitness. For many types of viruses, synthetic biology offers the potential to create virtually any genome order while maintaining wild-type sequences within all elements. Not all rearrangements necessarily reduce fitness, but many do, and those provide a means to attenuation. The attenuating effect of viral genome rearrangement was first demonstrated for vesicular stomatitis virus (Wertz, Perepelitsa, and Ball 1998; Ball et al. 1999) and has since been shown for a few other viruses (Endy et al. 2000; Flanagan et al. 2001); it was shown even earlier for some bacteria (reviewed in Rocha 2008). The engineered fusion of segments in segmented viruses poses a similar means of attenuation (Onodera et al. 1998; Pena et al. 2013), as does the swapping of packaging signals in segmented viruses (Lowen et al. 2005).

### 7.1 Prediction

Predicting the degree of attenuation from a rearrangement requires an understanding of how the order of genome elements affects a major fitness component, which could be through effects on assembly, replication, transcription, translation, or others. The most advanced understanding of this sort is for the bacteriophage T7: a virtual model of the viral life cycle has been developed, and this model was used to predict the fitness effect of translocations of the phage RNA polymerase (RNAP) gene (Endy et al. 2000). T7 is unusual among DNA phages in that it encodes its own RNAP, which in turn drives expression of most of its genome. The linear entry of the T7 genome into its host requires expression of the RNAP; a ‘downstream’ shift in the location of the RNAP gene causes well-understood delays in the life cycle, giving rise to robust predictions of fitness drops. For most viruses, however, the fitness effect of a translocated element is not obviously as predictable nor prone to incremental modification as with silent codon changes.

Predicting the evolutionary stability of a rearranged genome is also problematic in all systems. The impact of rearrangements on viral growth rate is subject to change through mutations affecting gene expression, so it is feasible that some recovery mutations will have large effects and lead to rapid fitness gains despite an initially strong attenuating rearrangement that itself is irreversible.

### 7.2 Empirical tests

Various types of genome rearrangements have been generated in viruses. Some rearrangements have no obvious effects (Novella, Ball, and Wertz 2004); in others, the effects are pronounced (Onodera et al. 1998; Endy et al. 2000; Novella, Ball, and Wertz 2004; Lowen et al. 2005; Pena et al. 2013). In some cases, candidate vaccines have been developed (Flanagan et al. 2001; Pena et al. 2013). There is yet no suggestion of generalities that may be used to predict reliable attenuation by this method other than the general observation that rearrangements are often attenuating.

The most integrated analyses of attenuation by rearrangement are with T7 (Endy et al. 2000; Springman et al. 2005; Cecchini et al. 2013). For three different translocations of the RNAP gene, model predictions of viral fecundity rate (burst size per time) were only approximately in agreement. The most extreme displacement of the RNAP gene had a severe fitness impact, and this outcome was in broad agreement with the model. In another study with a rearranged T7, the presumed mechanism of life cycle retardation by RNAP gene displacement was tested by engineering an ectopic promoter predicted to rescue; the rescue failed. Given the deep understanding of T7 genome expression and the apparent simplicity of the relationship between genome entry, gene expression, and fitness, the difficulty in prediction raises doubts about the feasibility of prediction with other viruses.

**Figure 4.**
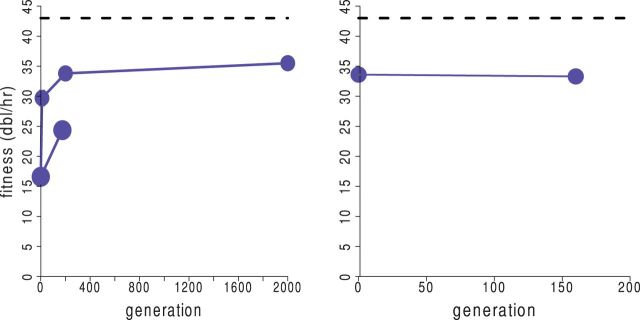
Evolution of attenuating genome rearrangements in phage T7. Blue curves give fitness (growth rate measured as population doublings/hour) across the generations of adaptation; the horizontal dashed line gives the approximate upper limit of fitness as observed in wild-type T7 adapted to the growth conditions. (Left) Two replicates of a genome with an RNAP gene displaced approximately half the genome length. In the replicate that evolved the higher fitness, an early rearrangement returned the RNAP gene to its wild-type location but displaced other genes. Subsequent evolution was gradual and may have reached an asymptote. (Right) The RNAP gene was displaced about 15% of the genome length, so the initial fitness was higher than in the left graph. However, no apparent fitness improvement occurred during adaptation. Both panels are from Cecchini et al. (2013), the left amended with data from Springman et al. (2005). Adapted with permission from Cecchini et al. (2013)

Only three studies have addressed the evolutionary stability of attenuation by rearrangement, all with bacteriophages. An unpublished study with a ϕ6 virus whose three segments were fused showed no fitness increase over 180 generations (Hanley, pers. comm. of work done by Hanley, Madert, and Chao). In T7, several rearrangements involved simple translocations of the phage RNAP gene; one involved an exchange of early and late genes that left the RNAP gene intact (Springman et al. 2005; Cecchini et al. 2013). Different rearrangements showed different patterns of early fitness recovery, some substantial, but all maintained a significant level of fitness depression on long-term adaptation (Fig. 4, even out to 2,000 generations). The T7 virtual model and accompanying biochemical information were not sufficient to predict details of the evolutionary response in most cases. Despite the difficulty in prediction, these studies raise the possibility of using genome rearrangements to create and evolve a permanently attenuated virus—something not obviously possible with codon modification.

Genome rearrangement is more easily introduced into some viruses than others. The polyprotein generated by poliovirus, for example, may limit the types of rearrangements possible. It is conceivable that other constraints imposed by small genomes (such as overlapping reading frames) will also limit the feasible types of rearrangements.

## 8 Attenuation by deletion: surprising recoveries

Perhaps the most obvious synthetic approach to attenuation is deletion of genomic elements. Deletions are feasible in a live vaccine only if the deleted elements are not essential for growth. All genes are essential in many viruses with small genomes, so it might seem that those viruses are not candidates for this means of attention. Yet even with small genomes, non-essential portions of the genome can be identified, perhaps within genes. With several possible targets for deletion in a single genome, the combinatorial possibilities for attenuated genomes may be large, and many of those combinations may need to be tested empirically to find acceptable reductions in fitness. A concern with any deletion is a possibly altered immune response due to the absence of antigenic epitopes.

Deletions may be used in two contexts. One is simply to reduce the growth rate of the virus, attenuating by reducing viral titer in the same way that the codon-deoptimization methods work (Whatmore et al. 1995; Berkhout et al. 1999; Licis, Balklava, and Van Duin 2000; Rokyta et al. 2002; Springman et al. 2005; Licis and van Duin 2006; Harcombe, Springman, and Bull 2009; Mazel-Sanchez and Elliott 2012); any non-essential gene or gene segment will potentially work. For example, the codon-deoptimized influenza virus of Nogales et al. (2014) targeted the NS1 gene, whose role is to defend against the host interferon response; it might be guessed that a deletion or partial deletion of NS1 would have a similar effect (as pointed out by a reviewer). A second is to selectively remove genes that are essential for virulence but not essential for viral growth (Enjuanes et al. 2008; Lamirande et al. 2008). The absence of such genes may of course impair viral growth, but the effect on virulence is for reasons other than growth rate. A well-known example from outside viruses comes from pathogenicity islands in bacteria, responsible for specific disease symptoms yet not necessary for bacterial growth (Groisman and Ochman 1996). (In a third approach, a virus may be deleted of a gene essential for growth or transmission and the vaccine be prepared with a complementing gene so that the vaccine infects and possibly replicates but is unable to produce infective progeny. This latter design ensures $R_{0}=0$, but as those viruses are dead ends, they are considered no further.)

### 8.1 Prediction

There are few generalities evident. The fitness-reducing effect from deleting a non-essential element is usually difficult to predict even semi-quantitatively without a detailed model of genome dynamics. Indeed, one of the surprises of bacteria and yeast genetics is that many genes can be deleted without detectable fitness effects (Nichols et al. 2011; Giaever and Nislow 2014). However, regardless of the fitness effect of a deletion, different deletions together should often lead to greater fitness reduction than do the individual deletions, enabling a coarse- or moderate-scale tuning of fitness reduction. Where the goal is to delete non-essential virulence determinants, the predicted degree of attenuation will again rest on the details of the system, which includes both the host response and the virus.

The ‘obvious’ prediction about evolutionary recovery is that there can be no recovery of a viral genome carrying a deletion, since evolution is not likely to recreate the deleted portion (some exceptions are noted below). Yet even when the deletions persist, their fitness effects may be ameliorated by evolution in genes to restore the balance of processes affected by the deletion. Prediction thus requires not only an understanding of the interacting partners and processes of the missing element but also of how those partners may compensate. If the deletion is of an element that functions only to cause disease, the predicted absence of virulence recovery may be on safer grounds.

### 8.2 Empirical tests

As is evident from the preceding discussion, the method of engineering deletions to attenuate viral fitness is decades old. The method commonly succeeded, but the magnitude of attenuation needs to be evaluated empirically. More surprisingly, evolutionary recovery of genomes with deletions poses a surprisingly serious problem to sustained attenuation. Some evolutionary recoveries of small deletions in RNA viruses have been recreations of near-original sequences (Whatmore et al. 1995; Licis, Balklava, and Van Duin 2000; Licis and van Duin 2006). In an HIV-1 with three partial deletions, duplication in a promoter region led to major fitness increases while leaving intact the original deletions (Berkhout et al. 1999).

One of the best studied evolutionary recoveries from the perspective of underlying mechanisms is of DNA ligase gene deletions in the related phages T7 and T3 (Rokyta et al. 2002; Harcombe, Springman, and Bull 2009). Phage DNA ligase is ‘essential’ when the virus is grown on a host whose DNA ligase is also defective, but sustained adaptation led to massive fitness increases in both viruses and to approximately the same final fitness. In T7, the observed compensatory mutations were primarily in DNA metabolism genes, whereas in T3, the compensatory changes were both in DNA metabolism and in genes unrelated to DNA metabolism. The fact that a reduction in genome replication (by disruption of DNA metabolism) creates an imbalance in the different processes of viral assembly allows most any gene to interact with, and thus compensate for, the absence of ligase.

The cases of extreme recoveries from deletions is certainly disturbing, at least if the basis of attenuation is fitness reduction. Offsetting this concern is the fact that some recoveries were slow, at least initially, though some were not. Thus, deletion-based vaccines that are intrinsically capable of extensive recoveries might die out before they evolve the ability to persist. Furthermore, recoveries that leave the deletion intact are typically only partial, a point that led Berkhout et al. (1999) to propose a stepwise method of attenuation. Viruses engineered with deletions would be evolved for recovery, then engineered to carry other deletions, followed by a second recovery. The process would be extended as needed, allowing quantitative attenuation and an endpoint of stable, permanent attenuation.

## 9 Discussion

Considerable progress in genetic engineering technology gives us the ability to engineer nearly any viral sequence of choice and thus to implement nearly any imaginable design for attenuated vaccines. The limiting step is now our understanding of what attenuates, and for viruses capable of autonomous amplification, how evolution can reverse that attenuation. Several approaches made feasible by technology have been tested enough, if only in culture, to get a sense of the repeatability and hence the predictability of the process. The emphasis here is on approaches that are potentially general to many viruses and have also been implemented at least experimentally. Designs specific to one virus are sometimes used and may work well, so they may warrant consideration on a case-by-case basis.

No method is yet sufficiently predictable to avoid the need for experimentation. The method offering the greatest promise of *a priori* predictable attenuation, based on regularity between fitness decline and modification, is silent codon change. But some viruses show unexpected irregularities. Predictability should improve as more is understood about how codon changes attenuate; the recent proposal that attenuation is due to CpG and UpA dinucleotides may lead to a profound improvement in prediction. In contrast, attenuation by genome rearrangement or deletion poses a much greater challenge to prediction, and difficulties were experienced in a system considered highly amenable to prediction.

Evolutionary recovery is also difficult to predict. Silent codon modification is sometimes characterized by the expected slow recovery, but the agreement is at best qualitative and not general to all viruses tested. Furthermore, the method does not appear to enable ‘permanent’ attenuation. Evolutionary recovery in response to deletions and rearrangements is often large despite retaining the genomic disruptions attributed to attenuation, but there are suggestions that both methods might lead to quasi-permanent attenuation after initial phases of evolutionary recovery. Permanent attenuation opens the door for many types of passive vaccination programs that require little ongoing public health effort.

Once experience has been obtained with a particular vaccine and it has proven robust against evolutionary reversion, it may be possible to use that stable genome for vaccines against other serotypes, by replacing key antigens. This approach has been implemented in the Flu Mist influenza vaccine (Flu Mist fact sheet, 2015, at http://www.medimmune.com/docs/default-source/default-document-library/product-and-patient-information-for-flumist-quadrivalent.pdf?sfvrsn=0). The approach avoids the need to attenuate each serotype, but it does require *a priori* knowledge of antigens driving the immune response.

Kenney et al. (2011) offered an interesting evaluation of alternative methods. They engineered three different designs in the same virus and then evaluated attenuation and reversion in side-by-side comparisons during serial transfers in mice. The three designs consisted of the traditional attenuation method (adaptation to novel conditions), a rationally engineered strain with a nonsynonymous point mutation in an envelope gene and a deletion, and a chimera between virulent virus and an avirulent relative. After 10 mouse–mouse transfers (by intracerebral injection), the chimeric virus exhibited the greatest stability of attenuation. This experimental, comparative approach has obvious advantages in discovering suitable designs and even helps avoid experimenter bias in becoming vested in a single approach. Of course, other studies and combinations of studies have compared different attenuation designs of the same virus but not so explicitly and directly as did Kenney et al. (2011).

Viral chimeras—recombinants incorporating genes from close or distant relatives—are widely used in research and offer several different possibilities for attenuation. The challenges of many chimeric vaccine approaches parallel those experienced by the methods discussed above so will not be discussed here. However, one chimeric technology is specific to chimeras and offers a unique possibility: changing viral receptors (Reimann et al. 2004; Michelfelder and Trepel 2009; Parrish 2010; Mourez et al. 2011). In extreme cases, altering the receptors can completely change the tissue tropism of a virus. In essence, use of a new receptor may endow the virus with a new niche. Depending on the dynamics of viral growth in the host, on the spectrum of mutations to other receptors, and on tradeoffs in alternative receptor use, a virus with altered tropism could be stably transmitted indefinitely under its new tropism. If this altered tropism is attenuating and also effects an appropriate immune response, a virus with a new and stable tropism may be permanently attenuated. This is an exciting area for original research, both theoretical (Kelly et al. 2003) and experimental.

One of the potentially biggest but unknown threats from the ultimate escape of live, attenuated vaccines comes from the few patients who establish persistent infections and continue transmitting for years. Adequate herd immunity will render this threat harmless, but the threat is especially important any time that vaccine coverage wanes or at the brink of eradication. If a live vaccine is not permanently attenuated, these patients must either be identified in advance and vaccinated with dead-end methods or they must be isolated post-vaccination until they are virus-free.

A factor in the evolutionary reversion of some attenuated vaccines is recombination. Recombination between an attenuated strain and the wild type may be inconsequential because it does no worse than reconstituting the wild type, which is already present in any host harboring the recombination. The problem gets messier when multiple wild-type strains are present and/or multiple strains are attenuated, because it now may be possible for a recombinant to acquire different properties than any of the parent strains and thus to have even higher fitness than circulating wild types. Of course, recombination among wild-type strains is not accelerated by the introduction of vaccine strains, but recombination among different vaccine strains or between vaccine and wild-type strains may be greatly increased in the high viral loads introduced by vaccination. The evolution of a high-fitness vaccine-recombinant strain has in fact been reported in a poultry virus, and the recombinant virus even appears to have higher fitness than the previous wild-type virus (Lee et al. 2012, 2015). When recombination poses a possible threat of this type, the vaccine design may be chosen to restrict opportunities for recombination or to restrict opportunities for the types of recombination that will be most pernicious. For example, silent codon modification may reduce sequence similarity so that recombination becomes improbable in certain segments. Attenuating deletions may also limit opportunities for creating viral recombinants whose fitness is higher than wild type.

Much existing work on evolutionary reversion of attenuated viruses has been done *in vitro*. It is still unknown how adaptations in tissue culture mirror the evolution that would occur in the actual host. Answering this question should be feasible with eukaryotic viruses that can be transmitted experimentally both in culture and in model organisms. Although fitness may not be comparable between culture and host, comparisons of the fitness trajectories and molecular evolution would be meaningful.
